# Optic Disk Pit and Iridociliary Cyst Precipitating Angle Closure Glaucoma

**DOI:** 10.5005/jp-journals-10008-1158

**Published:** 2014-01-16

**Authors:** Sushmita Kaushik, Parul Ichhpujani, Savleen Kaur, Surinder Singh Pandav

**Affiliations:** Associate Professor, Glaucoma Services, Advanced Eye Centre, Postgraduate Institute of Medical Education and Research, Chandigarh, India; Assistant Professor, Department of Ophthalmology, Government Medical College Chandigarh, India; Senior Resident, Glaucoma Services, Advanced Eye Centre, Postgraduate Institute of Medical Education and Research, Chandigarh, India; Professor, Glaucoma Services, Advanced Eye Centre, Postgraduate Institute of Medical Education and Research, Chandigarh, India

**Keywords:** Optic disk, Pit, Angle closure glaucoma.

## Abstract

Primary angle closure glaucoma is usually a bilateral disease, though it may be asymmetrical. However, it is unusual to see advanced glaucoma in one eye and no disk damage in the other. We present a case of unilateral angle closure glaucoma complicated by an optic disk pit and iridociliary cysts.

**How to cite this article:** Kaushik S, Ichhpujani P, Kaur S, Pandav SS. Optic Disk Pit and Iridociliary Cyst Precipitating Angle Closure Glaucoma. J Current Glau Prac 2014;8(1): 33-35.

## CASE REPORT

A 40-year-old woman presented with headache and diminution of vision in the right eye since 4 months. There was no systemic problem. The patient had been diagnosed as glaucoma in the right eye elsewhere and started on topical latanoprost 0.005% HS 1 month prior to presentation. Her records from the referring doctor showed the IOP to be 26 mm Hg before starting treatment.

**Fig. 1:**
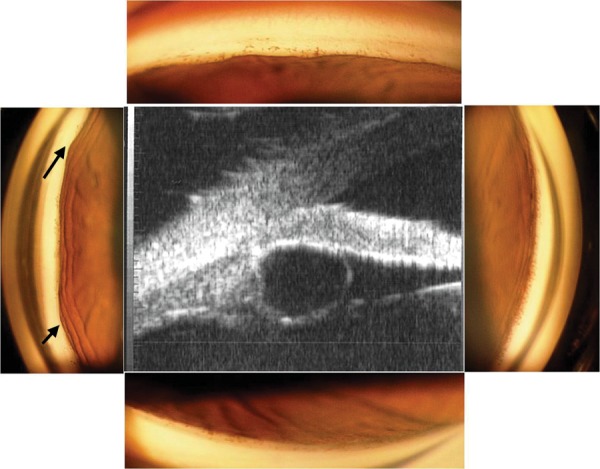
Gonioscopy of the right eye showing occludable angles with bumpy appearance of the peripheral iris (arrows). Note that the goniopictures shown have been taken under bright light to demonstrate the bumpy appearance of the iris, and does not refect the extent of closure seen with a narrow slit-lamp beam (center). UBM picture showing iridociliary cyst

The best-corrected vision was 6/24 and 6/6 in the right and left eye with +0.5 and +0.75 diopters respectively. The intraocular pressure (IOP) was 16 mm Hg in the right eye on topical latanoprost and 12 mm Hg in the left eye without treatment. The peripheral anterior chamber was shallow. The right eye angle was closed on gonioscopy with intermittent synechial closure and patchy pigmentation on the trabecular meshwork. The peripheral iris had a ‘bumpy' appearance suggesting iridociliary cysts or plateau iris configuration (Fig. 1). The left eye had occludable angles but it was an appo sitional closure with no peripheral anterior synechiae. She was clinically primary angle closure suspect (PACS) in this eye.

Iridociliary cysts in the right eye were confirmed by ultrasound biomicroscopy (UBM) (Fig. 1). The cysts were multiple of varying sizes, and extended intermittently in the superior, nasal and temporal angles, but not in the superior angle.

The axial length of the right and left eye was 21.14 and 22.33 mm respectively. The anterior chamber depth was 2.37 and 2.58 mm in the right and left eye respectively.

**Figs 2A to D:**
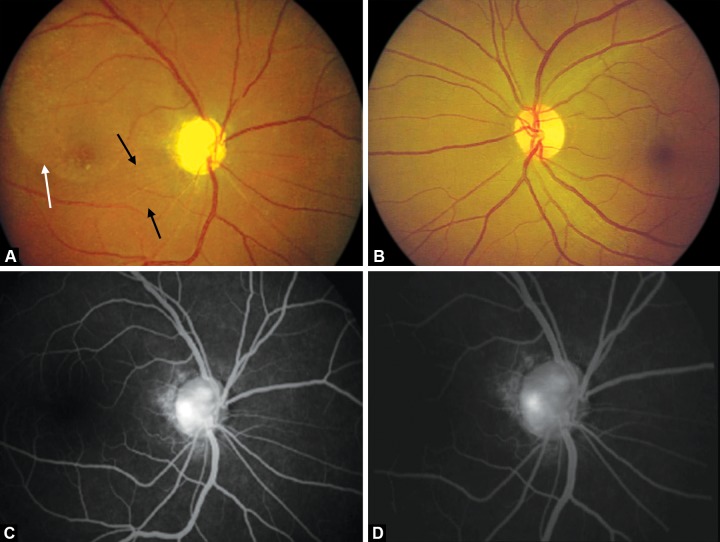
(A) Deeply excavated optic disk, superotemporal retinal detachment (white arrows) and ILM folds (black arrows). (B) Normal left eye. (C and D) FFA showing persistent staining of the temporal disk area suggestive of a pit

Posterior segment evaluation of the right eye revealed cup-disk ratio 0.9 with a deeply excavated cup (Fig. 2A). There was a serous retinal detachment supero-temporal to the macula, and internal limiting membrane folds extending from the disk to the macula. The left eye was normal (Fig. 2B). Fundus fluorescein angiography of the right eye showed persistent staining of the optic disk in the late phase localized to one area (Figs 2C and D), suggestive of an optic disk pit, but it was not evident clinically.

**Figs 3A to D:**
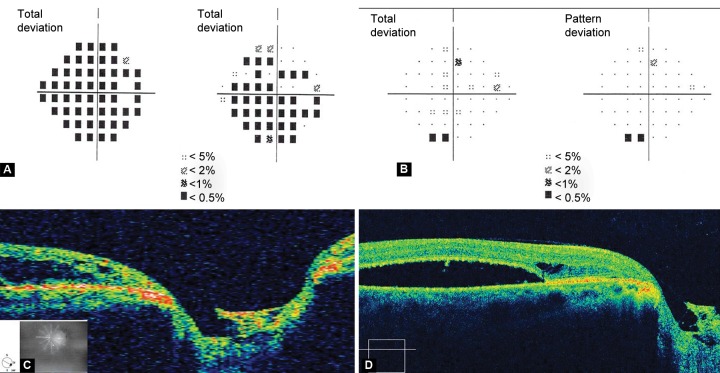
(A) advanced visual field defect in the right eye. (B) Normal visual fields in the left eye. (C) Stratus and (D) Cirrus OCT scan through the suspected area showing excavation in the lamina cribrosa and serous macular detachment

Visual fields showed advanced glaucomatous damage in the right eye and were normal in the left (Figs 3A and B). Time-domain optical coherence tomography (Stratus OCT) (Fig. 3C) showed an excavation in the lamina cribrosa suggestive of an optic disk pit. There was no communication between the retinal detachment and the pit in any scan. Findings on the spectral-domain OCT (Cirrus OCT) (Fig. 3D) were similar and no conduit could be detected.

Laser peripheral iridotomy was done in both eyes. In the right eye, the angles opened up in the areas of appositional closure, but the areas with PAS and the iris cysts remained closed. The IOP was 20.0 mm Hg without treatment, so she was started on timolol maleate 0.5% eyedrops after the LPI in the right eye. The left eye angles opened up and IOP was 16 mm Hg without treatment after the LPI. Follow-up after 3 months revealed an IOP of 16 mm OD (on timolol) and 14 mm OS.

### Comment

This patient illustrates how inherent conditions in the eye may lead to advanced optic disk damage in an eye with primary angle closure. Records of the referring doctor showed her IOP to be raised prior to starting treatment. The right eye iridociliary cyst could have contributed to an eye predisposed to angle closure and had probably led to angle closure glaucoma in this eye.^1^ The iridociliary cyst was picked-up on UBM after a careful gonioscopy raised a clinical suspicion of the possibility. The chronic angle closure in the right eye could have been aggravated by widespread presence of iridociliary cysts closing the angle in addition to primary angle closure. This would explain the asymmetry in the glaucoma status in both eyes.

The optic disk pit visualized on FFA and OCT was most likely congenital as per the location on the optic disk and the presence of the serous detachment.^2^ Acquired pits have usually been described in primary open angle glaucoma and normal tension glaucoma, more commonly seen in the center of the disk.^3^ It is possible that due to the congenital optic disk pit, the lamina cribrosa was weaker in this eye and led to more extensive damage despite the IOP not being too high. Congenital optic disk pits have been reported to have thinner RNFL furthering a postulation of increased susceptibility to glaucoma in these eyes.^4^ Optic disk pits have also been reported to be at increased risk for progressive optic disk damage and visual field loss in patients with glaucoma.^5^

The association of an optic disk pit and iridociliary cyst with primary angle closure glaucoma has not been previously reported. The iridociliary cyst explains the extent of synechial closure in the right eye and the presence of optic disk pit explains the severity of glaucomatous nerve damage. Such a constellation of signs might explain the asymmetric angle closure glaucoma with advanced disk damage in one eye seen in our patient.
